# Mesenchymal stem/stromal cell therapy for COVID-19 pneumonia: potential mechanisms, current clinical evidence, and future perspectives

**DOI:** 10.1186/s13287-022-02810-6

**Published:** 2022-03-24

**Authors:** Weiqi Yao, Lei Shi, Yun Zhang, Haibo Dong, Yu Zhang

**Affiliations:** 1 Department of Hematology, Union Hospital, Tong Ji Medical College, Hua Zhong University of Science and Technology, Hubei, China; 2 State Industrial Base for Stem Cell Engineering Products, No. 12 Meiyuan Road, Tianjin, 300384 China; 3 Hubei Engineering Research Center for Human Stem Cell Preparation, Application and Resource Preservation, Wuhan, China; 4 Department of Infectious Diseases, Fifth Medical Center of Chinese, PLA General Hospital, National Clinical Research Center for Infectious Diseases, Beijing, China; 5 Tianjin Key Laboratory for Stem Cell and Regenerative Medicine, Tianjin, China; 6 Wuhan Optics Valley VCANBIO Cell & Gene Technology Co., Ltd., Hubei, China; 7 Tianjin Key Laboratory for Blood Cell Therapy Technology, Tianjin, China

**Keywords:** MSC, Cell therapy, COVID-19

## Abstract

The coronavirus disease 2019 (COVID-19) pandemic, caused by severe acute respiratory syndrome coronavirus 2 (SARS-CoV-2), has spread into more than 200 countries and infected approximately 203 million people globally. COVID-19 is associated with high mortality and morbidity in some patients, and this disease still does not have effective treatments with reproducibly appreciable outcomes. One of the leading complications associated with COVID-19 is acute respiratory distress syndrome (ARDS); this is an anti-viral host inflammatory response, and it is usually caused by a cytokine storm syndrome which may lead to multi-organ failure and death. Currently, COVID-19 patients are treated with approaches that mostly fall into two major categories: immunomodulators, which promote the body’s fight against viruses efficiently, and antivirals, which slow or stop viruses from multiplying. These treatments include a variety of novel therapies that are currently being tested in clinical trials, including serum, IL-6 antibody, and remdesivir; however, the outcomes of these therapies are not consistently appreciable and remain a subject of debate. Mesenchymal stem/stromal cells (MSCs), the multipotent stem cells that have previously been used to treat viral infections and various respiratory diseases such as ARDS exhibit immunomodulatory properties and can ameliorate tissue damage. Given that SARS-CoV-2 targets the immune system and causes tissue damage, it is presumable that MSCs are being explored to treat COVID-19 patients. This review summarizes the potential mechanisms of action of MSC therapy, progress of MSC, and its related products in clinical trials for COVID-19 therapy based on the outcomes of these clinical studies.

**Supplementary Information:**

The online version contains supplementary material available at 10.1186/s13287-022-02810-6.

## Background

Severe acute respiratory syndrome coronavirus 2 (SARS-CoV-2) [1] has caused a global pandemic of coronavirus disease 2019 (COVID-19) which has an R0 value that is similar to Spanish influenza but higher than the Middle East respiratory syndrome (MERS) and H1N1 influenza [2]. Recently, several SARS-CoV-2 variants, especially the highly transmissible delta (B.1.617.2) and the immuno-evasive lambda (B.37) have caused a second wave of pandemics in many countries [3, 4]. Until August 31, 2021, more than 202,608,306 COVID-19 cases had been confirmed globally with a mortality rate of 2.1% based on a report by the World Health Organization (WHO) (www.WHO.int). COVID-19 has a variety of symptoms, ranging from the mild, moderate, severe, and critically severe [5]. According to the WHO report, the vast majority of COVID-19-positive people developed only mild (40%) or moderate (40%) symptoms; approximately 15% of COVID-19 patients developed severe symptoms that needed oxygen support, and 5% of patients developed critically severe symptoms with complications such as acute respiratory distress syndrome (ARDS), respiratory failure, sepsis and septic shock, and/or multiorgan failure (including cardiac and acute kidney injury). ARDS is an anti-viral host inflammatory response that is usually caused by cytokine storm syndrome (CSS) which may lead to multi-organ failure and has become the leading cause of death in severe and critically severe COVID-19 patients. CSS, which is triggered by SARS-CoV-2, involves a variety of inflammatory cytokines such as tumor necrosis factor (TNF), IL-2, IL-6, IL-7, MIP1A, granulocyte colony-stimulating factor, interferon gamma-induced protein 10, and chemokine (CC motif) ligand (CCL) family members [6, 7]; CSS is closely linked to COVID specific ARDS, multi-organ failure, and eventual death [8–10].

Currently, there is no cure for COVID-19. According to WHO guidelines, severe and critically severe COVID-19 patients should be administered anti-inflammatory and anti-viral drugs in addition to supportive therapies such as invasive and non-invasive mechanical ventilation [11]. To date, various drugs have been tested in clinical trials for their safety and efficacy, including in two major categories: (1) anti-viral drugs such as remdesivir [12], lopinavir-ritonavir [13], favipiravir chloroquine, and hydroxychloroquine [14]; and (2) immune-modulators such as anakinra, an IL-1 receptor antagonist [15], tocilizumab and sarilumab, both of which are IL-6 receptor antagonists [16], and ruxolitinib and baricitinib, which are Janus kinase signal inducer pathway inhibitors [17]. In addition to the aforementioned two major categories of treatments, other methods, such as neutralizing antibodies [18, 19] and convalescent plasma therapy [20, 21], are also used in the clinical fight against COVID-19. However, none of the above-mentioned drugs/treatments can significantly and reproducibly improve the symptoms of COVID-19 patients and are thus not recommended by the WHO for COVID-19 treatment. Recently, the emergence of several COVID-19 variants, especially the delta variant that has caused rapid infection in India and other countries, provides an urgent impetus to find an effective therapy for COVID-19, especially for severe and critically severe patients [22, 23]. More recently, mesenchymal stem/stromal cells (MSCs), the multipotent stem cells that exhibit both virus-resistant and immunomodulatory activity, and that can differentiate into a variety of cell types (as well as its derivatives), have been used to treat COVID-19 patients in the clinic owing to their immunomodulatory and tissue repair functions [24, 25]. This review focuses on the potential mechanisms of actions of MSC therapy, progress of MSC, and its related products in clinical trials for COVID-19 therapy based on outcomes of clinical studies.

## MSCs as a potential therapy against COVID-19

**Fig. 1:**
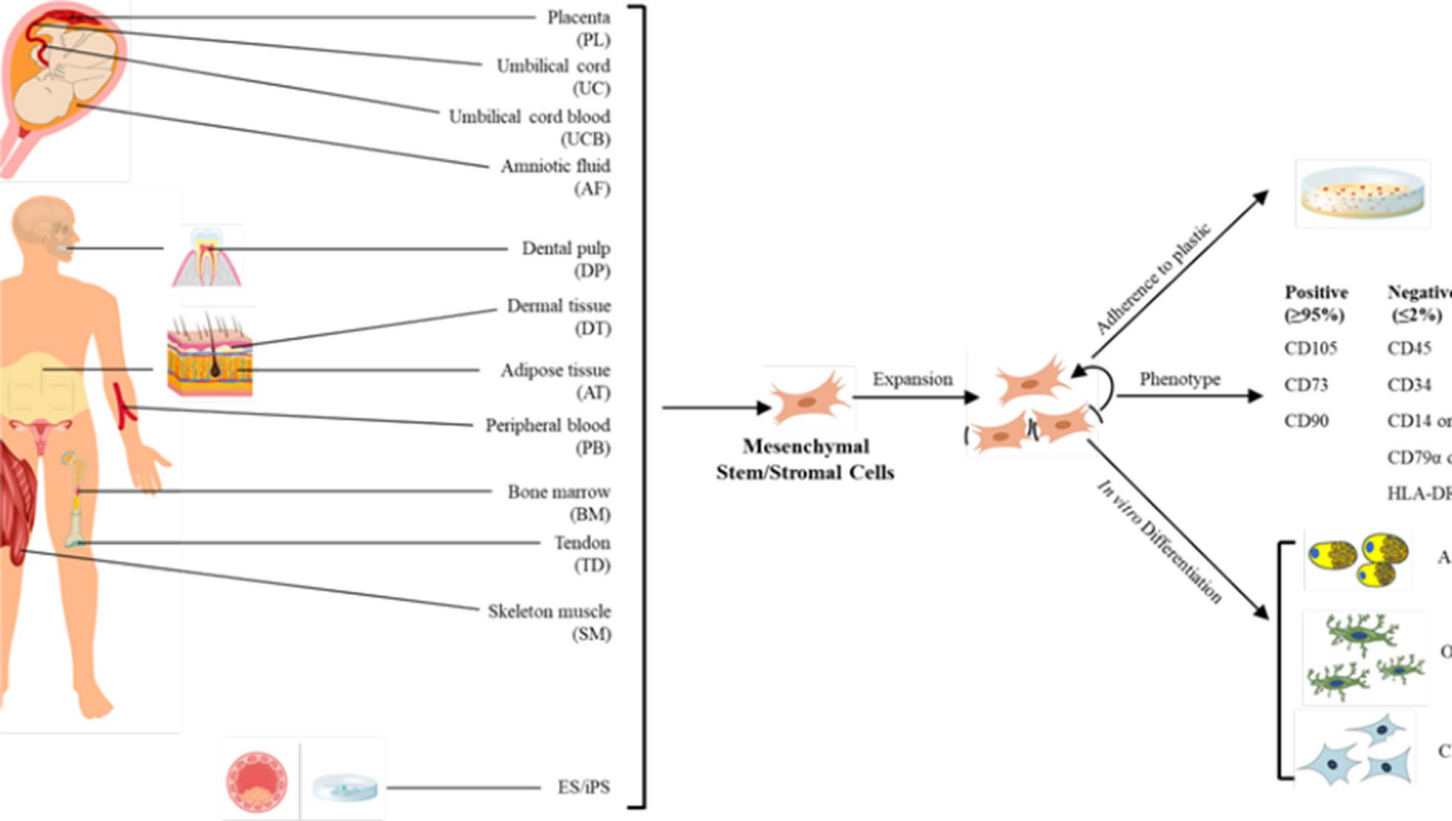
Sources and minimal characteristic criteria of MSCs

MSCs are multipotent stem cells that may be isolated from several adult tissues including adipose tissue (AT-MSCs), bone marrow (BM-MSCs), skin, dental pulp (DP-MSCs), foreskin, salivary gland, and fetal tissues including amniotic fluid, umbilical cord (UC-MSCs), placenta, Wharton’s jelly, and cord blood (Fig. 1) [26, 27]. MSCs are characterized by: (1) their capacity to adhere to plastic surfaces, (2) their expression of CD105, CD73, and CD90, and their lack of expression of CD45, CD34, CD14 or CD11b, CD79α or CD19, and HLA-DR surface molecules, and (3) their capacity to differentiate into multiple cell lineages including adipocytes, osteoblasts, and chondroblasts in vitro differentiating conditions [28], all of which fit the definition of stem cells as suggested by the International Society for Cell & Gene Therapy (ISCT). MSCs have been widely used for improving immune dysfunction and for facilitating damaged tissue regeneration [29]. Both autologous and allogeneic BM-MSCs, UC-MSCs, and AT-MSCs [30] are applied in the clinic due to the low expression levels of major histocompatibility complex class I and the near absence of major histocompatibility complex class II on their surface [31]. Given the current extensive application of MSCs in the clinic, it is presumable that MSCs may be used to treat COVID-19 patients with a compromised immune system, damage of organs such as the lung, and ARDS.

## Possible mechanisms of MSCs underlying COVID-19 treatment

**Fig. 2:**
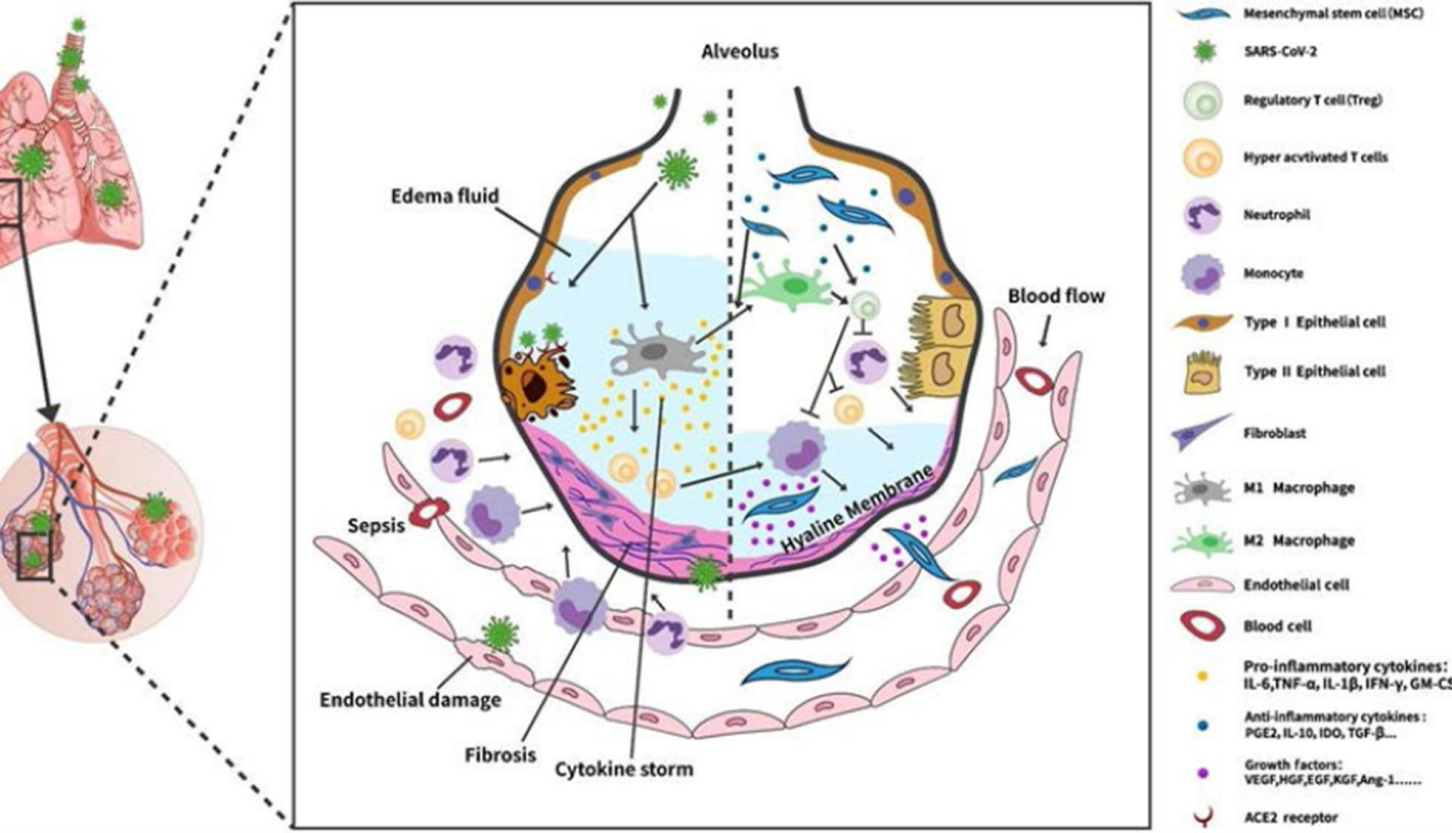
Potential mechanisms of action of MSCs in the treatment of COVID-19 pneumonia. MSCs regulate the COVID-19-triggered cytokine storm and lung damage through its immunomodulatory and trophic functions. MSCs can secrete various anti-inflammatory cytokines, e.g., PGE2, TGF-β, IDO, and IL-10, to promote the differentiation of macrophages from the pro-inflammatory type M1 to the anti-inflammatory type M2, to reduce the neutrophil infiltration, and to regulate hyper-activated T cells. On the other hand, MSCs can secrete growth factors to inhibit fibrosis and to suppress epithelial/endothelial cell apoptosis and influx of alveolar fluid

In advanced stages of COVID-19 infection, CSS can induce ARDS, pulmonary edema, dysfunctional air exchange, cardiac injury, and even death. CSS also occurs in graft-versus-host disease during graft failure and leukemia or lymphoma in response to CD19 CAR-T therapy [32]. CSS in COVID-19 often occurs with a median time of 8–14 days in an average of 15% of the infected patients [33]. Stimulated by the pro-inflammatory factor such as TNF-α [34], MSCs secrete trophic and immunomodulatory factors as mentioned above, MSCs express their functions mainly through paracrine effects, i.e., secreting immunomodulatory cytokines such as indoleamine 2,3-dioxygenase (IDO), prostaglandin E2 (PGE2), IL-6, and IL-10 to balance pro-and anti-inflammatory responses [35–38], as well as growth factors such as vascular endothelial growth factor (VEGF), hepatocyte growth factor (HGF), platelet-derived growth factor (PDGF), insulin-like growth factor 1, and fibroblast growth factor 2 (FGF2) which promote cell regeneration and angiogenesis following tissue injury [39, 40]. These mechanisms support the notion that MSCs may reduce or even eliminate CSS among COVID-19 patients. On the other hand, MSCs can also be regulated by cell-to-cell contact, and they can secrete extracellular vesicles [41]. These mechanisms support the notion that MSCs may reduce or even eliminate CSS of COVID-19 patients. The detailed possible mechanisms of action of MSCs are described below (Fig. 2).

### Anti-inflammation

During inflammation, impaired epithelial cells, the major barrier component of blood vessels and tissues, increase the permeability of lung tissues [42]. Previously, intratracheal administration of MSCs in lipopolysaccharide-induced inflammatory conditions in a mouse model has been shown to reduce inflammation and injury-increased permeability of the lung tissues by inducing IL-10 through secreting PGE2, granulocyte–macrophage colony-stimulating factor (GM-CSF), and granulocyte colony stimulating factor (G-CSF) [31]. Additionally, MSCs release anti-inflammatory factors IL-10 and IL-4 to repress the activation of lymphocytes and inflammatory cytokines such as IL-1-α-β,, -6, -17, and TNF-α [43]. On the other hand, MSCs prevent infection-induced damage to lung tissues by decreasing the excessive secretion of neutrophil extracellular traps at the infectious site [44]. During bacterial infections, MSCs reduces inflammation and ameliorates tissue injury through at least the following mechanisms: (1) by diminishing the excessive production of neutrophils and enhancing neutrophil-mediated phagocytosis [45], (2) promoting macrophages to differentiate into M1, which induces phagocytosis and promotes bacterial clearance, and M2, which benefits tissue repair by attenuating inflammation at the infection site [46, 47], and (3) promoting the proliferation of regulatory T cells and inhibiting the proliferation of effector T cells, thereby diminishing the immune response and ameliorating lung damage in ARDS [48].

MSCs can also attenuate inflammation-linked tissue injury through the regulation of transcriptional responses and protein–protein interaction. In a septic mouse model, MSCs were able to induce transcriptional responses via upregulating the nuclear factor of activated T cells calcineurin gene family members that mediate the expression of cytokine genes and downregulating Toll-like receptor-mediated nuclear factor kappa light chain enhancer of activated B cells [49]. In an acute lung injury mouse model induced by lipopolysaccharide, BM-MSCs were able to generate a physical contact with connexin 43, a gap junction protein, through which MSCs released mitochondria-containing micro-vesicles into alveolar epithelial cells. This transfer increased the amount of ATP in epithelial cells, thereby promoting the repair of alveolar endothelial and epithelial barriers in acute lung injury [50]. In addition, a study on an Escherichia coli-induced pneumonia model showed that mitochondrial transfer from MSCs to macrophages, which occurs in part through nanotube-like structures, enhances phagocytic activity and establishes a mechanism for anti-microbial effect through cell-to-cell contact [51].

As mentioned above, MSCs can perform functions through a paracrine activity. In a rat lung injury model that was generated by a ventilator, the conditioned medium collected from an MSC culture reversed the lung injury via keratinocyte growth factor (KGF), which ameliorates the epithelial cell injury by potentiating the activity of Na-KATPase, anti-inflammatory cytokine (matrix metallopeptidase 9, IL-1α), and GM-CSF [52, 53]. Overexpression of some factors released by MSCs, such as EGF, PDGF-b, angiogenin 1, and basic FGF, increases cell proliferation and facilitates lung repair [54]. Studies from different research groups also suggested that the overexpression CXCR4, angiogenin 1, ACE-2, KGF, and HGF attenuates endotoxin-triggered lung injury, collagen deposition, and fibrosis and edema formation, in part by enhancing the anti-inflammatory and chemotactic properties of MSCs [55–58].

### Immunomodulation

It has been shown that MSCs modulate the immune system during lung injury that is induced by respiratory viruses, which may also be one of the mechanisms underlying the COVID-19 treatment by MSCs. For example, in a mouse model of avian influenza virus (H5N1)-induced lung injury, UC-MSCs recovered the function of alveolar epithelial cells, as evidenced by reduced permeability and elevated alveolar fluid clearance [59]. In addition, the function of MSCs is not significantly affected by viral infections, which may be partially attributed to the fact that intrinsically expressed interferon-stimulated genes (ISGs) prevent viruses from “attacking” MSCs because the induction of intrinsic ISGs in human MSCs triggers the expression of anti-viral factors including SAT1, PMAIP1, ISG15, IF16, CCL2, and interferon-induced transmembrane protein 1 (IFITM1) [60].

The IFITM family members are important protectors and prevent several viruses, including Rift Valley fever virus, Ebola virus, influenza A virus, dengue virus, reovirus, and SARS-CoV from entering cells through the lipid bilayer [61, 62]. Interestingly, the host cell receptor ACE-2 in IFITM-expressing cells prevents the internalization of SARS-CoV viruses [61]. In the lungs, the ACE-2 receptor is expressed in endothelial and alveolar type II cells, and these cells contribute to blocking virus entry and diminishing fibrosis and thus exhibiting endothelial-protective and anti-inflammatory functions [63–65]. The potential benefits of overexpression of ACE-2 receptors in MSCs in COVID-19 treatment warrants further exploration.

### Cell death prevention

Regulated cell death (RCD), such as apoptosis, pyroptosis, necroptosis, and autophagic cell death, plays an important role in injury of tissues and organs, including lung [66]. While appropriate levels of regulated cell death may help tissue regeneration by removing damaged cells and reduce the accumulation of toxins released by injured cells and thus are appreciated, exacerbated regulated cell death may heighten inflammatory response and promote tissue injury [67]. Histological examination of postmorten lungs of COVID-19 patients uncovered the presence of apoptosis and necroptosis [68], suggesting the contribution of regulated cell death to lung injury in COVID-19 patients. Interestingly, one of the major functions of MSCs is to prevent RCD [69]. For example, ARDS patients who received MSCs treatment and had significant improvements also had significant decrease in the levels of cell death [70]. However, the direct evidence that links MSCs treatment and decrease in cell death in COVID-19 patients remains to be provided, although MSCs may counter regulated cell death through anti-inflammatory and immunomodulatory actions as mentioned above.

While it is well known that MSCs possess multiple biological functions, exact mechanisms behind the treatment of COVID-19 patients by MSCs remain to be elucidated.

## Clinical experience and outcomes of treatment of viral infections and respiratory diseases with MSCs: lessons from the past

MSCs have been extensively applied to treat infectious and non-infectious diseases because of their regenerative and immunomodulatory activity.

### Hepatitis B virus (HBV)

Chronic HBV infection is a primary cause of severe liver diseases, especially in East Asian populations, and liver transplantation is the sole cure for the end-stage liver diseases such as acute liver failure and decompensated liver cirrhosis [71]. BM-MSCs was shown to be resistant to HBV infection [72], and the administration of both autologous BM-MSCs and allogenic UC-MSCs in liver failure patients caused by HBV significantly increased the survival rates, elevated the circulating levels of serum cholinesterase, albumin, platelet, prothrombin, and decreased serum levels of alanine aminotransferase and total bilirubin [73, 74]. Consistent with the above observations, Wang et al. also reported that UC-MSCs were well-tolerant and increased the survival rate of patients with chronic HBV induced liver failure and cirrhosis [75]. Recently, after the long-term follow-up of 75 months, the MSC-treated group showed significantly improved liver function and a higher overall survival rate than the control group while there was no significant difference in the hepatocellular carcinoma event-free survival rate between these two groups [75][75]. However, to further clarify the safety and efficacy of MSCs in treatment of HBV-induced severe liver disease patients, double-blind, placebo control, multi-center randomized clinical trials with a long-term follow-up period are needed in the future.

### Avian influenza virus (AIV)

AIV, such as H7N9, is another potential threat in terms of a global pandemic of a respiratory tract infectious disease. H7N9-infected patients usually develop ARDS, acute pneumonia, and lung failure, which are similar to the complications of COVID-19 patients. Little is known from a clinical perspective regarding whether MSCs can be safe and effective for treatment among H7N9 patients. In a recent open-label clinical trial at a single center, Chen et al. transplanted allogeneic MB-MSCs into 17 patients with H7N9-induced ARDS and 44 patients with H7N9-induced ARDS were included as a control group. Notably, the MSCs treatment group had a significantly lower mortality rate than the control group. It is noteworthy that MSCs transplantation did not cause any harm to patients during the five-year follow-up period [77].

### Human immunodeficiency virus (HIV)

Very few clinical studies were performed that use MSCs to treat HIV patients. In an open-label study, Zhang et al.[78]. treated seven non-immune responders (NIRs) patients with three doses of UC-MSCs, and the control group had six NIRs. They found increased levels of naive and central memory CD4+ T-cell as well as elevated production of IL-2 and HIV-1-specific interferon. Recently, Wang et al. reported that hUC-MSC treatment for NIRs with chronic HIV-1 infection was safe and well-tolerated [79]. In addition, this study revealed significantly increased CD4+ T counts in the low-and high-MSC dose groups after a 48-week treatment, compared with no change observed in the control group. Moreover, the cumulative probability of achieving an immunological response was higher than the control. However, there were no significant differences in the CD4/CD8+ T counts and CD4/CD8 ratio between these two groups [79].

### ARDS

ARDS exhibits high morbidity and mortality and lacks effective treatments. In a phase I clinical trial, MSCs were administered to patients with acute lung injury (ALI) and ARDS, and this trial demonstrated that MSCs from different sources were safe and well-tolerated in ARDS patients [80] with some patients showing improvement in the respiratory and hemodynamic function as well as outcomes regarding multiorgan failure [81]. A phase II randomized controlled trial (RCT) conducted in the United States demonstrated that a high dose (10 × 10^6^ cells/kg) of allogeneic BM-MSCs infusion did not cause any significant respiratory AEs [82]. Moreover, this high dose of BM-MSCs infusion improved the oxygenation index and reduced the circulating levels of ANG-2 in these patients, which suggests ameliorating endothelial injury [82].

## Current clinical trials of MSCs and its products in treatment of COVID-19

To date, more than 90 clinical trials have been registered at ClinicalTrials.gov that use MSCs to treat COVID-19. Among these MSCs used, UC-MSCs accounted for 35%, followed by BM-MSCs (20%), AD-MACs (15%), and DP-MSCs (7.5%) [83–85]. MSCs are usually administrated intravenously a single time or multiple times, and the doses range from 0.5 × 10^6^ to 5 × 10^6^ cells/kg per injection. Currently, more than 65% of these trials are in phase I/II or II, and approximately, 22% are in the early phase or phase I; only a few (less than 5%) are in phase II/III or III. The detailed information of clinical trials is listed in Additional file 1: Table S1.

Several MSC products have also been used in the above-mentioned clinical trials, and some of them are in the phase II or III stage. Mesoblast in collaboration with Novartis initiated a double-blind, randomized, and placebo-controlled phase III trial with 300 patients using an intravenous infusion of 2 × 10^6^ cells/kg BM-MSCs after encouraging results had been obtained from 12 ventilator-dependent ARDS patients [86–88]. A completed phase I/II clinical trial of intravenous injection of MultiStem in COVID-19 patients has shown appreciable findings: a phase I study confirmed safety with a small starting dose, and a phase II study on 36 patients was a randomized, double-blind, and placebo-controlled trial. Compared to the placebo group, the treatment group had lower mortality and a shorter stay in the intensive care unit without any adverse effects [89, 90]. Athersys is currently carrying out a phase II/III clinical trial to examine the safety and efficacy of the BM-MSC product MultiStem in 400 COVID-19 patients with ARDS. Hope Biosciences is currently performing three clinical trials using autologous and allogenic AD-MSCs to evaluate its safety and efficacy in COVID-19 patients [91]. Pluristem has conducted a phase II double-blind, placebo-controlled, multi-center RCT study with 140 patients using intramuscular injection (300 × 10^6^ cells) of placenta-derived mesenchymal-like cells after a previous finding of the full recovery of six severely ill COVID-19 patients [92]. Cynata has initiated an open-label, RCT with an iPSC-derived MSC product Cymerus in 24 patients in the intensive care unit. MSCs are derived from iPSC-differentiated mesenchymal angioblasts, which were generated by transgene-, viral-and feeder-free techniques with the reprograming of donated blood cells [93]. Novellus and Citius proposed a placebo-controlled randomized dose escalation trial to assess the safety and efficacy of NoveCite in COVID-19 patients with ARDS. NoveCite is a product of MSCs derived from induced pluripotent stem cells, which are reprogrammed from fibroblasts with messenger RNA [94]. The detailed information of clinical trials with MSC products is listed in Table 1.

**Table 1:** MSC products in COVID-19 treatment

	Product	Company	Source	Dose	Phase	Country	Trial ID NO
1	VUM02	VCANBIO	Allogenic UC-MSCs	4E7 cells, i.v. at days 0, 3, and 6	I II	China	NCT04252118 NCT04288102
2	RYONCIL™(remestemcel-L)	Mesoblast, Ltd./Novartis	Allogenic BM-MSCs	2E6 cells/kg, 2 times/week	III	USA	NCT04371393
3	Multistem	Athersys, Inc	Allogenic BM-MSCs	–	II/III	USA	NCT04367077
4	HB-adMSCs	Hope Biosciences	Autologous AT-MSCs	5 times, i.v	II	USA	NCT04349631
5	HB-adMSCs	Hope Biosciences	Allogenic AT-MSCs	2E8 cells, i.v. at weeks 0, 2, 6, 10, and 14	II	USA	NCT04348435
				1E6 cells, at days 0, 3, 7, and 10	II	USA	NCT04362189
6	PLX-PAD	Pluristem Ltd	Allogenic placenta MSCs	3E8, i.v	II	USA, Germany, & Israel	NCT04389450 NCT04614025
7	JadiCell™	Therapeutic Solutions International	Allogenic UC-MSCs	(10 ± 2) E7 cells; at days 0 and 3	I/II	USA	NCT04355728
8	CYP-001 (Cymerus MSCs)	Cynata Therapeutics Ltd	iPSC-MSCs	2E6 cells/kg, i.v	I/II	Australia	NCT04537351
9	NestaCell	Cellavita	iPSC-MSCs	2E7 cells i.v. at days 1, 3, 5, and 7	II	Brazil	NCT04315987
10	HCLM051	Healios/Athersys, Inc	Allogenic BM-MSCs	9E8 (± 20%) cells	II	Japan	NCT03807804
11	itMSCs	Stemedica Cell Technologies, Inc	Allogenic BM-MSCs	–	II	USA	NCT04780685
12	ACT-20	Aspire Health Science	Allogenic UC-MSCs	1E6 cells/kg, i.v	I/II	USA	NCT04398303
13	*i*-MSC	Citius Pharma/Novellus	iPSC-MSCs	–	II	USA	–
14	CAStem	Zephyrm Biotech	ESC-MSCs	3, 5 or 10 E6 cells/kg, i.v	I/II	China	NCT04331613
15	ULSC-CV-01	Restem, LLC	Allogenic UC-MSCs	1E8 cells, i.v	I/IIa	USA	NCT04494386
16	COVI-MSC	Sorrento Therapeutics, Inc	Allogeneic AT-MSCs	1E6 cells/kg or 1.5E6 cells/kg, depending on CRP level	II	USA	NCT04728698
				3E7 cells at days 0, 2, and 4	II	USA	NCT04903327 NCT04905836
				1.85E7 cells at days 0, 2, and 4;3.7E7 cells at days 0, 2, and 4	Ib	USA	NCT04909892
17	BX-U001	Baylx Inc	Allogenic UC-MSCs	Low dose: 0.5E6 cells/kg; Middle dose: 1.0E6 cells/kg; High dose: 1.5E6 cells/kg	I/IIa	China	NCT04452097
18	PSC-04	Sorrento Therapeutics, Inc	Allogenic AT-MSCs	–	I	USA	NCT04486001
19	BM-Allo.MSC	ImmunityBio, Inc./NantKwest	Allogenic BM-MSCs	–	Ib	USA	NCT04397796
20	MB-MSC injection	IPM Biotech	Allogenic MB-MSCs	9E7 cells, i.v. at days 1, 3, and 5	I	China	ChiCTR2000029606
21	LMSCs	Longeveron Inc	–	1E8 cells, i.v. at days 0, 3, and 6	I	USA	NCT04629105
22	Descartes-30	Cartesian Therapeutics	Allogenic UC-MSCs	–	I/II	USA	NCT04524962
23	SB1-101	Sentien Biotechnologies, Inc	Allogenic MB-MSCs with an FDA-approved plasmapheresis device	High dose: 7.5E8; Low dose: 2.5E8	I/II	USA	NCT04445220
24	AlloRx™	Vitro Biopharma/GIOSTAR	Allogenic MSC engineered to secrete human DNases	–	I	USA	–
25	DW-MSC	Daewoong Pharmaceutical	Allogenic UC-MSCs	5E7 cells, i.v	I	Indonesia	NCT04535856
26	hDP-MSC injection	SH Bio-Tech	Allogenic DP-MSCs	3E7 stem cells at days 1, 4, and 7	I	China	NCT04336254

i.v., intravenous injection

## Current outcomes of clinical trials of MSC in COVID-19 treatment

Clinical experience with MSC treatment for COVID-19 is still limited. Although many clinical trials have been registered in clinicaltrials.gov (Additional file 1: Table S1), only a few have reported their findings (Additional file 1: Table S2). In China, MSCs have also been initiated as a therapeutic strategy for COVID-19, which was shown in several case reports. For example, the injection of human UC-MSCs into a 65-year-old woman on ventilation decreased the circulating levels of C-reactive protein (CRP), serum bilirubin, and liver function enzymes, and increased the circulating levels of CD8+ T, CD3+ T, and CD4+ T cells to the normal level [95]. This patient eventually recovered and tested negative for the virus [95].

As mentioned above, most MSCs used in the reported clinical research were derived from the umbilical cord. In one clinical trial [96], UC-MSCs were injected intravenously into seven COVID-19 patients (two mild, four severe, and one critically severe) with a dose of 1 × 10^6^ MSCs/kg, and three severe COVID-19 patients administered with the placebo served as controls. This trial was followed up for 14 days. The treatment group significantly improved lung function, which was accompanied by a decrease in the levels of serum CXCR3+ CD4+ T cells, CRP, CXCR3+ CD8+ T cells, CXCR3, and NK cells, and an increase in the levels of regulatory dendritic cells. Furthermore, ACE-2 or transmembrane protease serine 2 was not expressed in MSCs. Shu et al. conducted a clinical trial in which 12 COVID-19 patients received hUC-MSCs treatment, and 29 patients were in the control group [97]. This study showed that the hUC-MSC group had no patients with a progression from severe to critically ill and zero deaths during the 28-day study period, while the control group had four patients with a 10.34% mortality rate. Also, the treatment group had a shorter time for clinical improvement and lung inflammation absorption as revealed by CT imaging. In addition, the treatment group had significantly lower levels of CRP and IL-6 after three days of infusion and faster time for the lymphocyte count to return to a normal level [97]. Guo et al. demonstrated that MSC therapy restored oxygenation, elevated PaO2/FiO2 and lymphocyte count, downregulated cytokine storms, decreased levels of serum CRP, procalcitonin, D-dimer and IL-6, without AEs after infusion of UC-MSCs into 31 severe COVID-19 patients [98]. Feng et al. revealed that intravenous infusion of UC-MSCs partially recovered pulmonary function through forced expiratory volumes in 1 s, and it improved the quality of life as revealed by the St. George's Respiratory Questionnaire, which indicates relatively long-term safety and preliminary efficacy for severe COVID-19 patients after a 3-month follow-up [99]. Recently, a Turkish group reported that UC-MSC treatment was safe in 210 severe/critically severe COVID-19 patients with 1–2 × 10^6^/kg infusion. The treated group had a significantly higher survival rate; they were accompanied by restored oxygenation such as SaO_2_ parameters and downregulated cytokine storm [100].

MSCs from other sources also exhibited a similar benefit to treating COVID-19 patients. Spanish researchers used AT-MSCs to treat 13 COVID-19 patients, with two receiving a single dose, 10 receiving two doses, and one receiving three doses. Median number of cells per dose was 0.98 × 10^6^ AT-MSC/kg body weight. These researchers showed that the treatment improved immune cell profiling with no adverse effects observed [101]. Menstrual blood-derived MSCs were infused into 26 patients with a total of 9 × 10^7^ cells per infusion every other day for three times a day, and 18 patients received only concomitant medications as control. While there was no significant difference in the incidence of most AEs between these groups, the MSC group showed a significantly lower mortality rate, significant improvement in dyspnea, SpO_2_, and chest imaging results [102]. In another clinical trial, 27 COVID-19 patients who received an infusion of MSCs derived from hESCs had normal levels of all hematological and clinical parameters and tumor markers; none of these treated patients developed any abnormal responses/AEs that are associated with hESC-MSC therapy. Clinical improvements were also—observed in patients within 84 days after treatment with hESC-MSC therapy [103].

In addition to these case reports and clinical studies, three research groups have demonstrated outcomes from a more strictly designed phase I/II double-blind RCT. Giacomo et al. tested a single-center, double-blind, phase 1/2a, RCT of UC-MSC infusions in treatment of 12 COVID-19 ARDS patients compared with 12 patients who received two infusions of vehicle. Patients in these two groups received comparable standard care. There was no significant difference in infusion-associated AEs between these two groups, and no serious AEs that are linked to UC-MSC infusion were observed; this indicates the safety of UC-MSC infusions. UC-MSC treatment was associated with significantly improved patient survival, SAEs (SAE)-free survival, and time to recovery [104]. Ismail et. al. conducted a double-blind, multicentered RCT with 40 critically ill COVID-19 patients. Among these patients, 20 received an intravenous infusion of UC-MSCs with 1 × 10^6^/kg body weight, and 20 received 100 ml saline (0.9%) solution as the control. The UC-MSC group had a survival rate 2.5 times higher than the control group, and even 4.5 times compared to patients with comorbidities in the control group. The UC-MSCs infusion remarkably reduced the circulating levels of IL6 in the recovered patients, with no ADs observed [105]. Wang’s group reported two consistent studies: a phase I exploratory study with 18 COVID-19 patients and a phase II double-blind, placebo control, multi-centric randomized clinical study with 100 severe patients. In the phase I study, zero patients developed serious infusion-associated ADs in nine treated patients (four of them were severe, five were moderate). Two patients in the treatment group developed transient facial flushing and fever, and one had transient hypoxia. One patient in the treatment group needed mechanical ventilation compared with four in the control group [106]. In the phase II study, compared with the placebo control group which contained 35 patients, UC-MSCs administration in 65 patients significantly improved the whole lung lesion volume from baseline to day 28, decreased the proportion of solid component lesion volume, and increased 6-MWT [107]. There were no differences in AEs and serum levels of tumor markers at 12 follow-ups between these two groups (unpublished data). In line with the above findings, several clinical trials have suggested that MSC treatment greatly improved solid component lesion volume, reduced sleep difficulties, and improved daily activity compared to the placebo treatment [108–117]. In addition, the MSC treatment group had a significantly higher ratio of patients who had normal CT images at month 12 follow-up than the placebo group. Neutralizing antibodies were all positive with a similar median inhibition rate in both groups during 12 month follow-up [108–117]. The detailed outcomes of all these published clinical trials are listed in Additional file 1: Table S2.

## Perspectives

The persistent COVID-19 pandemic has prompted scientists and clinicians to explore effective treatments since a clear unmet medical need still exists for severe and critically severe patients. Although anti-viral molecules, immunomodulators, neutralizing antibodies, and plasma have been administered for COVID-19 patients with ARDS and other life-threatening conditions as treatments, very few of these approaches showed reproducibly appreciable efficacy. MSCs are considered to be a candidate for treating CSS and repairing damaged lung tissues due to their multiple potent activities, including anti-inflammation, immunomodulation, and ability to secrete soluble vesicles and multiple growth factors. Promising outcomes have been reported from ongoing clinical trials involving MSC treatment for COVID-19: (1) patients were safe and well-tolerated after treatment with MSCs that were generated from various sources with a wide range of doses, (2) improvements were observed in patients after MSCs treatment, such as through decreasing circulating levels of pro-inflammatory cytokines and laboratory parameters, better lung inflammation absorption, and (3) MSCs-treated patients had faster increase in SpO_2_, a shorter hospital stay, and a higher survival rate. However, some scientific and clinical questions remain to be addressed. For example, what are the exact mechanisms underlying the MSCs’ treatment of COVID-19 patients? Which source of MSCs is the best for this treatment? At which stage will COVID-19 patients have the best outcomes as a result of MSCs treatment? Are there any COVID-19 patients who should not receive MSCs treatment? Can MSCs treatment reduce the long-term residual adverse effects associated with COVID-19 infection? In addition, will the combinatory therapy of MSCs with other supportive drugs work better than the single use of each individual treatment? Apart from cell-based therapy, exosome vesicles, and secretome of MSCs can be considered as an alternative. However, limitations of these closed trials were also acknowledged. For example, most of these trials had a small sample size and were single-arm or parallel control; they lacked a strict design such as the double-blind, randomized, placebo control with multiple centers. Also, the main and secondary evaluation criteria were not uniform, and the long-term follow-up was not carried out. Currently, consensus and guidelines require doctors to better manage severely ill COVID-19 patients by using MSCs treatment [118]. For example, for subject selection, it is recommended to choose the patients with the most appropriate risk–benefit balance. In addition, the heterogeneity of subjects’ baseline conditions is also an important factor in determining the success or failure of clinical trials. For safety and efficacy evaluation, the first clinical trial is expected to explore the safety and tolerability of MSCs in patients, including exploring the maximum tolerated dose, observing the incidence, timing, and severity of expected or unexpected AEs that are correlated with cell infusion. It is not recommended to include patients with large differences in baseline and prognostic prediction in the same clinical trial. For risk control, the tumorigenicity should be continuously monitored t hrough long-term follow-up.

## Conclusions

The general mechanisms of action of MSCs include immunomodulation and tissue repair capability (antifibrosis and angiogenesis), and current preliminary clinical results of MSC-based therapies have shown some favorable outcomes for severe and critically severe COVID-19 patients, thus making it a promising therapy. However, double blind RCTs with large sample sizes are still required to thoroughly examine the safety and efficacy of MSCs and each specific MSC product. Nevertheless, MSC-based therapies during a global pandemic brings hope to combating COVID-19 and in meeting urgent medical needs, although a variety of challenges still lay ahead.

## Supplementary Information

**Additional file 1**. **Table S1.** Clinical trials of cell-based therapies for COVID-19 patients registered in clinicaltrials.gov/. **Table S2.** Current outcomes of clinical trials of MSCs in COVID-19 treatment.

## Data Availability

The datasets used and/or analyzed during the current study are available from the corresponding author on reasonable request.

## Abbreviations

ADs: AEs
AT: Adipose tissues
ALI: Acute lung injury
BM: Bone marrow
ESC: Embryonic stem cells
GI: Gingival tissue
IDO: Indoleamine 2, 3-Dioxygenase
IL-6: Interleukin-6
i.v.: Intravenous injection
MD: Mean difference
MOA: Mechanism of action
MSC: Mesenchymal stem/stromal cells
PBS: Phosphate buffered saline
SADs: SAEs
TGF: Transforming growth factors
TNF: Tumor necrosis factor
Treg: Regulatory T cells
UC: Umbilical cord
UCB: Umbilical cord blood
hES-M: MSC-like cells derived from human embryonic stem cells
P-MSCs: Placenta-derived MSCs
DP-MSC: Dental pulp-derived MSCs
MSC-EV: Extracellular vesicles derived from MSCs
POM-MSC: Pooled olfactory mucosa MSCs
MB-MSCs: Menstrual blood-derived MSCs
ARDS: Acute respiratory distress syndrome
PI: Pulmonary interstitial fibrosis
NR: Not reported
